# Do first-time avian migrants know where they are going: the clock-and-compass concept today

**DOI:** 10.3389/fphys.2025.1562569

**Published:** 2025-04-15

**Authors:** Nikita Chernetsov, Gleb Utvenko

**Affiliations:** ^1^ Ornithology Lab, Zoological Institute of the Russian Academy of Sciences, St. Petersburg, Russia; ^2^ Department of Vertebrate Zoology, St. Petersburg State University, St. Petersburg, Russia; ^3^ Biological Station Rybachy, Zoological Institute of the Russian Academy of Sciences, St. Petersburg, Russia

**Keywords:** animal navigation and orientation, bird migration, genetics of behavior, endogenous programs, clock-and-compass concept, vector navigation, innate beacons, innate component magnetic map

## Abstract

What if your life depended on finding a place you’ve never been—without a GPS device, a guide, or any way of knowing where to go? For young songbirds, this is the reality of their first migration. While this once puzzled researchers studying bird migration, advances in the field have since uncovered that many songbirds rely on an inherited genetic program to guide their remarkable solo journeys. Today, the most widely accepted theory explaining how young birds of species that migrate solitary and do not follow experienced conspecifics find their way to wintering grounds is the ‘clock-and-compass’ concept. According to this concept, naïve migrants follow a certain compass direction for a pre-defined period. In the simplest case, when the program runs out, they find themselves in their species-specific non-breeding range. However, recent research suggests that this process might be significantly more complex. New data indicate that first-time migrants may not have a complete map but rather a system of beacons. This system could be based, for example, on geomagnetic cues or other cues that help first-year birds navigate their location along the migration route. To date, a significant body of evidence has been gathered to revise the classic ‘clock and compass’ program. It is likely that first-time migrants of many species (although perhaps not all) are capable of varying degrees of location control based on innate information. The question of what data sources they use and how precise their control remains open for further investigation.

## Introduction

Billions of birds annually migrate between their breeding and non-breeding areas (Hahn et al., 2009). Most importantly, many of them show site fidelity to their natal and former breeding sites, and to non-breeding areas (Sokolov, 1997). The degree of spatial accuracy varies between species, is difficult to study, and is a subject of ongoing debate, but there is little doubt that most birds return to the areas that are small compared to the range of their migratory movements, and those that do not return and show nomadic behaviour do so for a reason and not because they fail to navigate.

More than 60 years ago, Gustav Kramer suggested the map and compass concept, which initially referred to homing pigeons but is applicable to all animals that move beyond their usual home ranges. According to this concept, to reach a certain goal located beyond the sensory perception range, a traveling animal (e.g., a migrating bird) needs (i) a positioning system (map), i.e., a mental representation of space at the scale it intends to travel, and (ii) a compass system that allows it to select and maintain a certain compass direction of travel (Kramer, 1953). It means that the question ‘how migrating animals find their way’ actually consists of two closely related but independent questions: What is the physical and sensory basis of the positioning system (map), and what is the physical and sensory basis of the compass system. Both these questions are a subject of active research, and in recent decades, significant progress has been made in both fields, even though important open questions remain (Mouritsen, 2018; Wiltschko and Wiltschko, 2023).

The map and compass concept leaves open the question of how juvenile migrants perform their maiden travel, when they migrate to the areas they have never been before. In some species, social interactions play an important role, with first-time migrants closely watching and following experienced conspecifics, related or unrelated ones (Chernetsov et al., 2004; Mueller et al., 2013; Loonstra et al., 2023; Fugate et al., 2024). However, individuals of many species migrate solitary, often at night, and without much interaction with conspecifics. It has been suggested that such species follow an innate spatio-temporal programme that has been termed a ‘clock-and-compass’ programme (Gwinner and Wiltschko, 1978) but would be more appropriately called calendar-and-compass programme.

**FIGURE 1:**
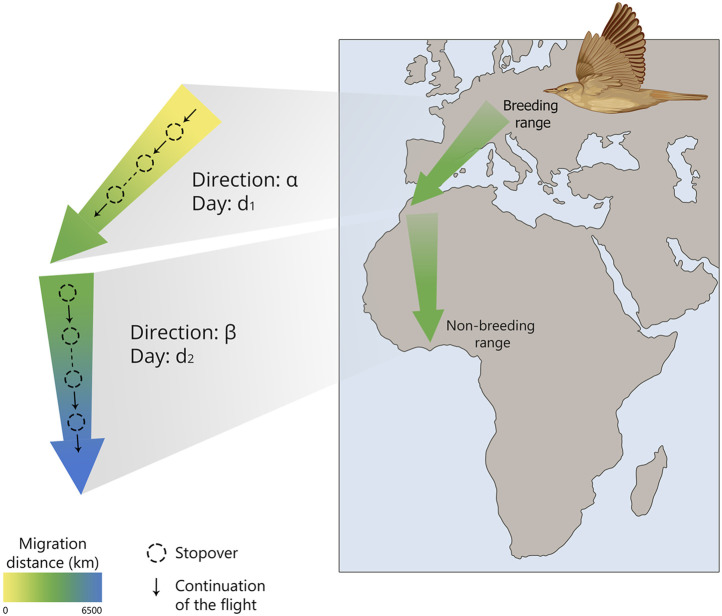
Schematic representation of the “Clock and Compass” concept in naïve migratory birds, showing how they navigate from their breeding grounds to their wintering areas using an internal timekeeping mechanism and directional cues.

Simplified, a young birds’ migration can be represented as a sequence of steps: begin migration at a certain age, head towards *α* and fly in that direction for *d*_*1*_ days, then change course to *β* and maintain it for *d*_*2*_ days, and so on. After several iterations, juvenile migrants reach their population-specific winter quarters (Figure 1). The change of migratory direction in a certain area is called *Zugknick* (from German, bend of migratory path). An obvious question is whether such a rather rough spatio-temporal programme is sufficient to perform migration to the population-specific wintering area. Weather events may delay migration, e.g., because of a protracted period of opposing winds, or a storm may blow the migrants off course. Obviously, such a programme is insufficient for pinpoint navigation, when migrants successfully reach a goal with linear size several orders of magnitude smaller than the range of migratory journey (several kilometres vs thousands of kilometres). This situation is typical of return migration when migrants get back to the areas where they have bred before or to their natal areas, showing breeding or natal site fidelity, or philopatry (Sokolov, 1981). However, also during first outward migration when first-winter individuals should reach their population-specific non-breeding area, with much more relaxed requirements for navigation accuracy (Cresswell, 2014), it is still not obvious that spatiotemporal programme without compensation for accidental movements off course would be sufficient, when the population-specific area is smaller than e.g., the whole of sub-Saharan Africa (Sokolov et al., 2024).

These considerations triggered hypotheses that assumed some kind of awareness of where migrating birds are currently located during the first migratory journey. These hypotheses received some support from experimental data.

### Clock-and-compass concept

**FIGURE 2:**
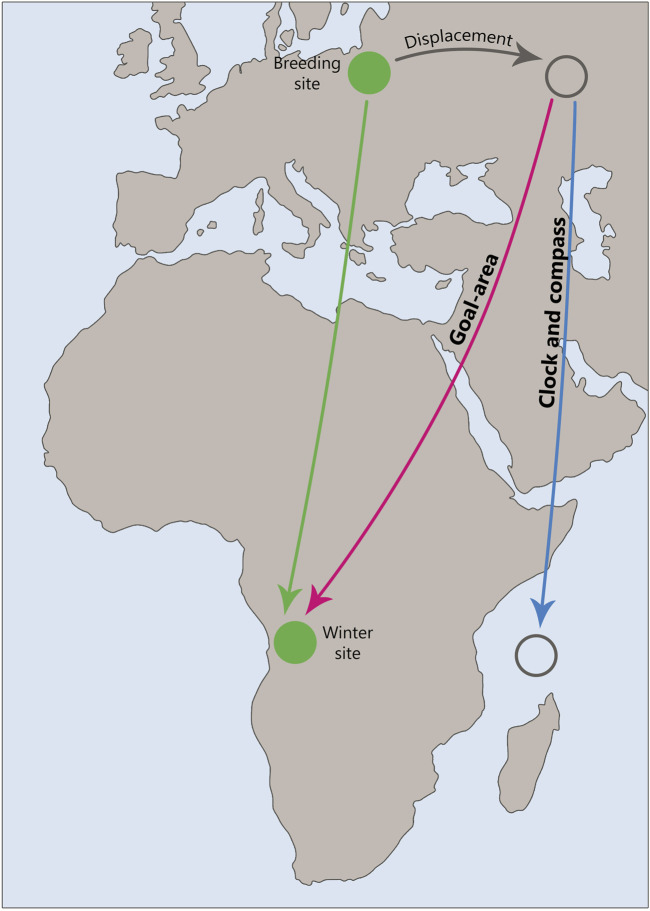
Schematic representation of a displacement experiment, illustrating the potential outcomes predicted by the “Clock and Compass” and “Goal-area Navigation” hypotheses. The diagram highlights the differences in navigational strategies and the resulting trajectories or goal areas in response to displacement.

The study of the concept of clocks and compass in young migrating birds through their physical displacement from their migration route (Figure 2) is important for understanding the mechanisms of navigation and orientation that these birds use during their long migrations. In this context, the compass is responsible for orientation, meaning the direction of movement relative to external factors such as the Earth’s magnetic field, the position of the sun, or the moving stars. The compass helps them understand which direction they need to fly. The question is whether birds following this inherited compass direction realize where they are currently, e.g., when they are displaced from the migratory route, e.g., by a storm or by the experimenter. This question is equivalent to the question whether they have some kind of a map.

Before the concept of clocks and compasses emerged, Rüppell (1944) displaced 507 hooded crows *Corvus cornix* 750 km west during their spring migration. The birds were transported from the Courish Spit on the southeastern Baltic coast to Flensburg in northern Germany, which is located north of their usual wintering sites. Ring recoveries showed that neither young nor adult birds compensated for the displacement. Instead, they settled parallel to the breeding range of the undisturbed individuals. The lack of compensation in hooded crows in this experiment was consistent with the clock-and-compass mechanism.

Subsequently, between 1948 and 1957, Perdeck (1958), Perdeck (1964), Perdeck (1967) conducted similar experiments, capturing 11,247 common starlings *Sturnus vulgaris* in the Netherlands and transporting them to Switzerland by airplane. According to the recoveries of the displaced birds, adult birds were able to correct their migration route and flew to the northwest towards their population-specific winter quarters in northwestern France and southern England, demonstrating their ability for navigation, meaning they used both the compass and the map. At the same time, young birds continued migrating to the southwest and remained for the winter in southern France and Spain, showing only the ability for orientation, that is, the use of the compass but not the map.

Based on these data, A. Perdek was one of the first researchers to suggest that young migrants follow a spatio-temporal program, while adults possess a bicoordinate map that allows them to correct for natural or artificial displacements. How important the studies by Perdeck remain to this day, is clearly illustrated by the recent publication of a re-analysis of his data (Pot et al., 2024). It showed that even though starlings are social and keep in groups outside of their breeding season, Perdeck’s results cannot be explained by his displaced birds joining the flocks of local conspecifics and following them.

**FIGURE 3:**
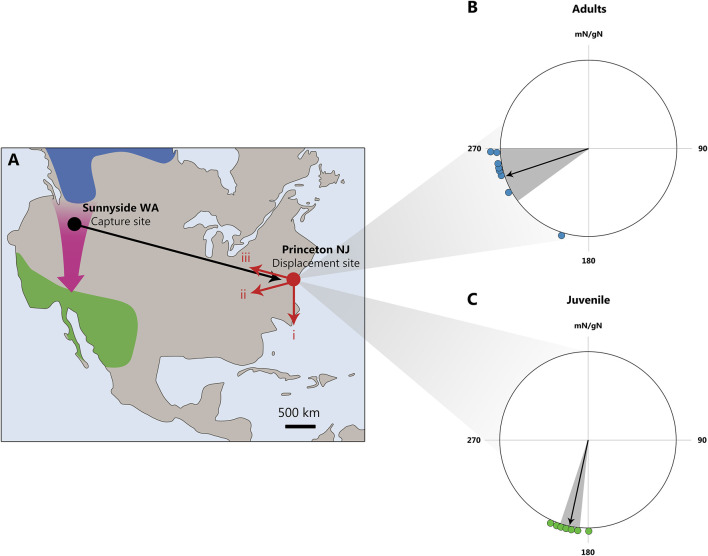
The displacement of white-crowned sparrows *Zonotrichia leucophrys* from Sunnyside, WA, to Princeton, NJ. **(A)** Map illustrating possible migration routes after release at Princeton: (1) continuation along the normal migration direction, (2) course correction toward the wintering area, and (3) return to the capture site. The breeding area is shown in blue, the wintering area in green, and the normal migration route with a purple arrow. **(B, C)**: Radio tracking data collected from small aircraft reveal that only adult, and not juvenile, long-distance migrating white-crowned sparrows were able to rapidly recognize and correct for a continent-wide displacement of 3,700 km. For both groups, only positions >25 km from the release site are included in the analysis. **(B)** Directions from the release site to the last observed positions for adults (blue). The mean direction = 252° ± 18°, r = 0.931 (Z = 6.94, N = 8, P < 0.001, Rayleigh test). One adult exhibited a southerly orientation, likely due to initial drift in strong northwesterly winds. **(C)** Directions from the release site to the last observed positions for juveniles (red). The mean direction = 192° ± 6°, r = 0.99 (Z = 8.82, N = 9, P < 0.001, Rayleigh test). Mean directions (black arrows) and 95% confidence intervals (gray area) are shown for adults and juveniles, respectively. mN/gN, magnetic North/geographical North. This figure is based on the results of Thorup et al. (2007).

Similar results were obtained by Kasper Thorup and co-authors in North America while studying the migration of white-crowned sparrows *Zonotrichia leucophrys* (Thorup et al., 2007). The researchers transported the birds from the northwest to the northeast of the United States (a physical displacement of 3,700 km) and used radio transmitters to track their movements after release. The authors found that birds that had already migrated were able to compensate for the longitude displacement, whereas young birds were did not do so (Figure 3).

Another example of age-related difference in migratory paths is demonstrated by streaked shearwaters *Calonectris leucomelas*. Shearwaters use a wind- and wave-based flight pattern, known as dynamic soaring, to extract energy for highly efficient travel over oceans, and therefore are generally reluctant to fly over land. However, whereas experienced adults circumvented the landmass of Japanese islands during their post-breeding migration towards the southwestern Pacific, juveniles flew across the landmass of Honshu Island, i.e., across most inhospitable terrain, apparently relying on an inherited compass direction (Yoda et al., 2017; Yoda et al., 2021).

The existence of a spatio-temporal program in birds was also confirmed in laboratory tests. Gwinner and Wiltschko (1978) showed that hand-reared garden warblers *Sylvia borin* from Germany followed an innate sequence of orientation behaviors when tested in circular arenas, which corresponds to the flight directions of their free-living counterparts. Garden warblers followed this sequence of predisposed flights in a specific compass direction, with their duration and timing being controlled by endogenous circannual rhythms. As a result, they reach the population-specific winter quarters.

**FIGURE 4:**
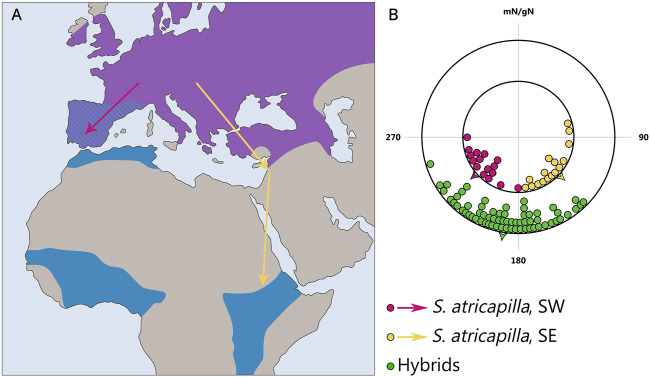
Inheritance of migratory direction in blackcaps *Sylvia atricapilla*: directional choices of hand-raised birds from SE- and SW-migrating populations during early autumn migration. **(A)** Map illustrating the breeding (violet) and primary overwintering (blue) areas of the blackcap. Arrows indicate the general autumn migration routes of the central European populations: southwest (yellow) and southeast (purple). **(B)** Directional choices of hand-raised blackcaps during the early and late parts of the autumn migration season. The inner circle represents the parental generation; purple dots indicate birds from the west Germany, and yellow dots represent birds from eastern Austria. The outer circle (green dots) shows the F1 generation. Arrowheads indicate the group mean migration directions. mN/gN, magnetic North/geographical North. This figure is based on the results of Helbig, (1991).

Furthermore, studies show that the spatio-temporal program is inherited and manifested in an intermediate form in first-generation hybrids with respect to both timing (for example, the duration of migration restlessness in blackcaps *Sylvia atricapilla*; Berthold and Querner, 1981; Berthold, 1988) and direction of migration (for example, studies on blackcaps (Figure 4), European robins *Erithacus rubecula*, blackbirds *Turdus merula*, and song sparrows *Melospiza melodia*; Helbig, 1991; Berthold and Helbig, 1992; Berthold et al., 1992). However, willow warblers *Phylloscopus trochilus* from central Sweden where two subspecies migrating towards the southwest and southeast intergrade, do not show an intermediate direction but migrate in either one or the other direction (Sokolovskis et al., 2023). Thus, the initial hypothesis of inherited migration direction was based on data about intermediate migration direction in hybrids, but further research has shown that the situation can be quite different [see a recent review by Caballero-López and Bensch (2024)].

**FIGURE 5:**
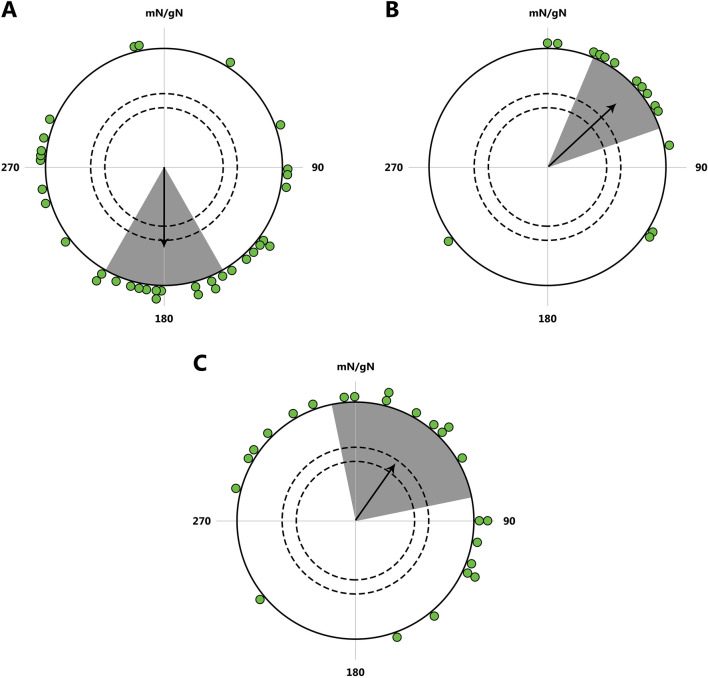
Orientation of naïve birds in Emlen funnels. **(A, B)**: Results obtained from juvenile pied flycatchers *Ficedula hypoleuca*. **(A)** Birds from 3 days of age were kept indoors without access to natural celestial cues, hand-fed, and tested in Emlen funnels in the fall, in a natural magnetic field and without access to stars. N = 35, mean direction 178° (r = 0.42, P = 0.002), 95% confidence interval (CI): 148°–209°. **(B)** Birds were kept indoors all winter without access to natural celestial cues prior to spring experiments were tested in Emlen funnels in the spring, in a natural magnetic field and without access to stars. N = 16, mean direction 47° (r = 0.70, P≪0.001), 95% CI: 23°–70°. **(C)** Results from juvenile Eurasian blackcaps *Sylvia atricapilla*. Birds were tested in Emlen funnels in the spring. N = 24, mean direction 35.4° (r = 0.395, P = 0.022), 95% CI: 352°–80.4°. The dots at the periphery of the circle represent the mean heading of a single bird, the arrow represents the group orientation mean vector (circle radius represents a vector length r = 1); the gray areas indicate the 95% CI; the inner and outer dashed circles indicate the required length of r for significance levels of 5% and 1% according to the Rayleigh test, respectively. mN/gN, magnetic North/geographical North. This figure is based on the results of Zolotareva et al., (2021); Wynn et al., (2023).

It has been shown that naïve songbirds, tested in Emlen funnels, exhibit innate seasonal spring orientation under laboratory conditions, despite never having undertaken their first migration and having spent the winter in the aviary (Figure 5; Zolotareva et al., 2021; Wynn et al., 2023).

Despite considerable advancements in molecular genetics and the development of research techniques over the past decade, the molecular mechanisms underlying migratory behavior in birds remain largely unexplained. While researchers have made notable strides in identifying genetic markers associated with migration, the specific genes and molecular pathways that regulate the complex processes of orientation, navigation, and migration timing are still not fully understood. Recent studies are beginning to shed light on this issue, revealing the role of specific genes (Bingman and Ewry, 2020; Chernetsov, 2023). One such gene is VPS13A, identified as a key element associated with the selection of wintering regions in songbirds such as the golden-winged warbler *Vermivora chrysoptera* and the blue-winged warbler *V. cyanoptera*. These species, which hybridize in the wild, have different migratory routes: blue-winged warblers winter in northern South America, while golden-winged warblers winter in both Central and South America (Toews et al., 2019). Variations in the VPS13A gene are closely linked to the selection of wintering regions and migratory direction. However, the role of this gene in birds is not yet fully understood, but it is hypothesized that it may influence energy turnover during migration. In addition, studies show that the VPS13A gene plays an important role in adaptation to different wintering conditions, allowing birds to successfully travel long distances and survive in various climatic zones. Although the link between this gene and migration has been established, the molecular mechanisms through which it influences bird behavior remain unclear. This discovery highlights the complexity of the interplay between genetic, physiological, and environmental factors influencing migration and necessitates further investigation.

A recent study conducted by Sokolovskis et al. (2023) revealed the genetic basis of autumn migration direction in willow warblers *P. trochilus*, marking a significant breakthrough in the study of orientation and navigation. In Scandinavia, two willow warbler subspecies breed: *P. trochilus* from southern Scandinavia and the Baltic coast, which winter in western tropical Africa and migrate towards the southwest, and *P. t. acredula*, nesting in northern Scandinavia and migrating south-southeast to winter in eastern Africa. These subspecies differ in size, plumage coloration, and genetic markers (Zhao et al., 2020; Caballero-López et al., 2022).

**FIGURE 6:**
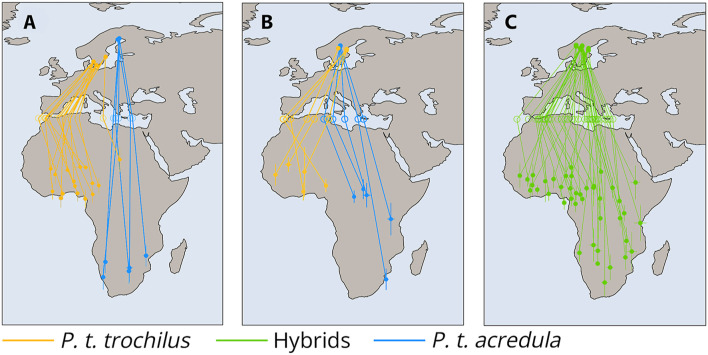
Migration routes and genetic differentiation of *P. t. acredula*, *P. t. trochilus* and hybrids across the Mediterranean Divid. **(A)** Tracks of allopatric *acredula* (N = 5) in blue and *trochilus* (N = 16) in orange. **(B)** Tracks of genetically defined acredula in blue (birds with the MARB-a allele and homozygous for *acredula* alleles on invP-Ch1 and invP-Ch5, N = 5) and genetically defined *trochilus* in orange (birds lacking the MARB-a allele and homozygous for *trochilus* alleles on invP-Ch1 and invP-Ch5, N = 5). **(C)** Tracks of birds from the migratory divide with hybrid genotypes (N = 41). Hollow circles in **(A–C)** indicate estimated longitudes where birds crossed latitude 35°N (Mediterranean Sea). The lines connect individuals to their respective breeding sites, Mediterranean crossing points, and primary wintering sites. Whiskers around locations in Africa in **(A–C)** represent standard deviations in longitude and latitude from the main winter site. This figure is based on the results of Sokolovskis et al., (2023).

Intrigued by this phenomenon, researchers discovered that the direction of migration in these birds is primarily determined by a combination of two genes: MARB-a and InvP-Ch1, with MARB-a suppressing the effect of InvP-Ch1 through epistatic mechanisms. This discovery is particularly important because it has shown that the migratory behavior of hybrids in the geographical regions where these subspecies overlap is inherited in such a way that their migration direction aligns with the parental populations’ migration patterns (Figure 6; Sokolovskis et al., 2023).

These findings have supported the hypothesis that the direction of bird migration can be inherited through complex polygenic mechanisms, suggesting that the spatio-temporal migration program can indeed be transmitted from generation to generation. However, the data also indicate that the mechanisms of inheriting migratory behavior may be species-specific. In some cases, hybrids inherit the migratory routes characteristic of their parental populations, while in others, they choose an intermediate direction, highlighting the diversity of genetic mechanisms underlying such behavior.

### Criticism of the clock-and-compass concept

At the moment, the ‘clock-and-compass’ concept is more or less the accepted mainstream of how migration proceeds in naïve migrants (Able and Bingman, 1987; Berthold, 1996; Åkesson and Helm, 2020). Avian migrants that strongly rely on social interactions may differ in this respect (Chernetsov et al., 2004; Mueller et al., 2013; Loonstra et al., 2023; Sokolovskis et al., 2024), but species whose migratory behavior does not strongly depend on social learning mechanisms are believed to follow a spatiotemporal program or clock-and-compass approach.

However, as often happens, after a mainstream view settles in, some ugly facts start to emerge that do not exactly fit in the pattern. In contrast to the “clock-and-compass” model, which explains bird orientation through the use of internal biological clocks and compass mechanisms, the concept of “navigation by goal area” suggests that migratory routes are determined by genetically encoded information about the geographic coordinates of destinations or stopover sites. This idea places inherited spatial targeting, rather than orientation based on directions or temporal parameters, at the core of migratory behavior, allowing birds to reach specific points regardless of their starting location. Rabøl (1978) analyzed recoveries of three passerine species in Europe and proposed the hypothesis that it is the geographic coordinates of wintering or stopover sites that are inherited. This approach challenges the traditional paradigm and highlights the need to reconsider established views on the nature of migration.

Further experiments conducted by Jørgen Rabøl over several decades demonstrated that young birds possess the ability to detect deviations from their route and, under certain conditions, adjust their course. These studies, based on artificial displacement of various bird species from their natural migratory paths, led to significant discoveries. Rabøl showed that migratory mechanisms include complex components that cannot be fully explained solely by the “clock-and-compass” model (Rabøl, 1969; Rabøl, 1970; Rabøl, 1972; Rabøl, 1975; Rabøl, 1981; Rabøl, 1985; Rabøl, 1993; Rabøl, 1994; Rabøl, 1995). He also argued that the results of Perdeck’s classic experiment conducted on a short-distance migrant (the starling) cannot be entirely extrapolated to long-distance migrants with more complex routes. Moreover, this author claimed that the results by Gwinner and Wiltschko (1978) may be interpreted differently (Rabøl, 1985). Data published several years after the original study, regarding migration routes in the Gibraltar area (Hilgerloh, 1989) and West Africa (Gatter, 1987a; Gatter, 1987b), suggest that the course change for most Palaearctic migrants occurs differently than what E. Gwinner and W. Wiltschko proposed for garden warblers. Apparently, the change in direction from a southwest to a southeast-east course does not occur after crossing the desert belt, but much later, along the northern coast of the Gulf of Guinea.

Studies have shown that migratory restlessness, typical of birds during the preparation for long flights, can be suppressed under experimental conditions. This is achieved by providing birds with unlimited food after a fasting period simulating the depletion of energy reserves during flight (Biebach, 1985; Gwinner et al., 1985; Gwinner et al., 1988). This scenario mimics natural conditions where birds reach suitable stopover sites, such as desert oases, to replenish their fat reserves. Notably, migratory restlessness disappears long before the birds achieve high fat levels and body mass. This suggests that the process of replenishing energy resources itself may serve as a signal for the temporary cessation of migratory behavior. Even when the total duration of such interruptions accounts for a significant proportion (25%–30%) of the autumn migration season, this does not lead to a proportional extension of the migration period. The migration season is not prolonged by the time spent at stopovers, despite the considerable time investment (Gwinner et al., 1992; Bairlein and Gwinner, 1994; Gwinner, 1996). If a similar situation occurs under natural conditions, and the duration of migration is indeed determined solely by an innate spatiotemporal program, some individuals may complete their migration prematurely, for instance, ending up in the middle of the Sahara. The consequences would most likely have been fatal.

Numerous studies on the impact of weather conditions on migration have confirmed that factors such as wind strength and direction significantly influence the migratory behavior of birds (Alerstam, 1978; Richardson, 1978; Richardson, 1990; Alerstam and Lindström, 1990; Åkesson, 1993a). In particular, unfavorable wind conditions can substantially increase energy expenditure and cause birds to deviate from their course. If young individuals during their first migration are unable to adjust their position and rely solely on innate directional knowledge and a temporal program, they risk failing to compensate for deviations and perishing.

Some published data suggest that at least in certain migratory bird species, young individuals undertaking their first migration possess if not a detailed navigational map, but at least a system of beacons enabling them to determine their position along the migration route at any given moment (Fransson et al., 2001; Thorup and Rabøl, 2007; Gschweng et al., 2008; Thorup et al., 2011; 2020). Very impressive in this respect are the circumpolar movements of juvenile Northern giant petrels *Macronectes halli* that are difficult to explain without assuming some kind of innate map (de Grissac et al., 2016).

Young individuals of some bird species indeed possess innate orientation abilities and elements of a navigational map. First-autumn Eleonora’s falcons *Falco eleonorae*, which migrate separately from adult birds, demonstrate rather complex species-specific migration routes. Their trajectories also exhibit individual characteristics, indicating a combination of innate mechanisms and environmental adaptations (Gschweng et al., 2008). The persistence with which juvenile falcons try to cross the Mozambique Channel and reach their species-specific winter quarters in Madagascar suggests that they have some innate knowledge of a landmass behind the water that they should reach.

**FIGURE 7:**
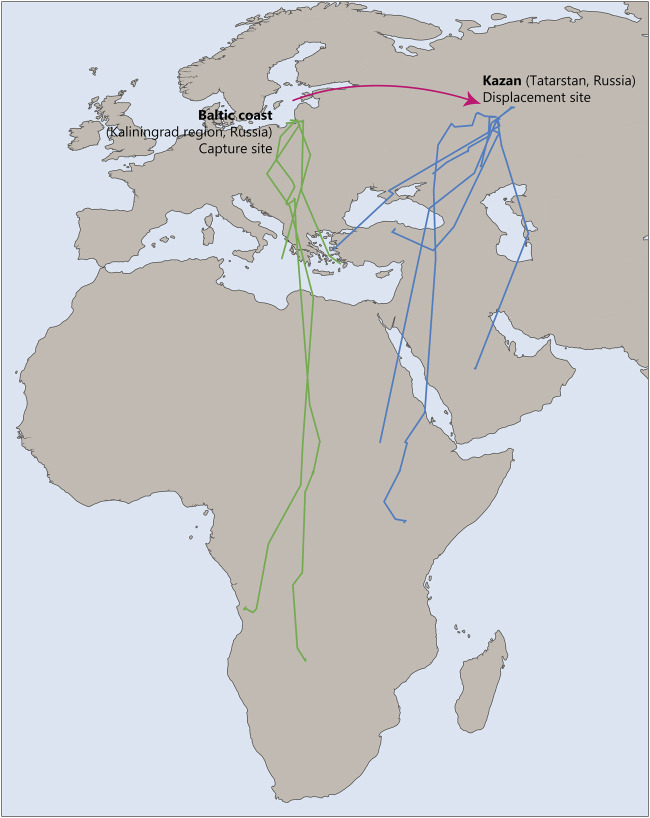
Tracks of control naïve common cuckoos migrating through Baltic coast, Kaliningrad region, Russia, during autumn (green) and experimental birds displaced to Kazan, Russia (blue). This figure is based on the results of Thorup et al., (2020).

Young common cuckoos *Cuculus canorus* also demonstrate the ability to compensate for deviations from their migration route. In a recent study common cuckoos were displaced 1800 km east from their original location on the Baltic coast to Kazan (Tatarstan, Russia; Thorup et al., 2020). The results suggested that young cuckoos were able to return to their species-specific migration route after such a displacement (Figure 7). The findings of this study are particularly interesting because it was previously believed that such corrective reactions were characteristic only of experienced birds. It suggests that their migratory behavior is based not only on an innate temporal program but also on genetically programmed spatial landmarks that ensure high accuracy in reaching necessary destinations, even with significant deviations.

**FIGURE 8:**
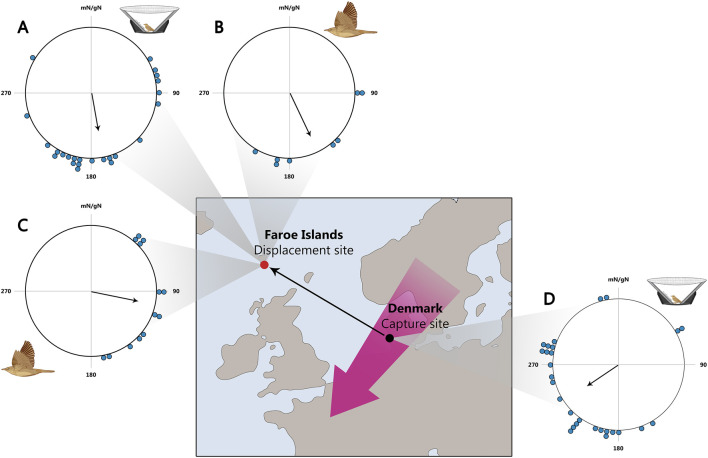
Orientation of naïve birds displaced to the Faroe Islands. **(A–C)**: Orientation on the Faroe Islands. **(A)** Tested in Emlen funnels of birds displaced from Denmark to the Faroe Islands. **(B)** Vanishing bearings of birds caught on the Faroe Islands. **(C)**: Vanishing bearings of birds translocated from Denmark to the Faroe Islands. **(D)** Orientation in Emlen funnels of birds caught and tested in Denmark. Map of Northwest Europe showing the location of the Faroe Islands (red dot) and the main migration through Northwest Europe (purple arrow). The 1,100 km displacement from Denmark (black dot) to The Faroes is shown by the thin arrow. In circular diagrams, the orientation of individual birds is marked on the periphery of the circle and the mean sample orientation is shown as a black arrow starting in the circle centre and with its length relative to the radius corresponding to the length of the mean vector r. mN/gN, magnetic North/geographical North. All sample orientations differ significant from random according to the Rayleigh test (P < 0.05). This figure is based on the results of Thorup et al., (2011).

Thorup and co-authors showed that young common whitethroats *Curruca communis*, garden warblers, and willow warblers are capable of compensating for shifts in their autumn migration route by more than 1,000 km (Figure 8). This was confirmed both under experimental conditions and through observations of birds that experienced natural displacement due to winds from Scandinavia to Faroe Islands (Thorup et al., 2011).

Thus, in our view, the concept of ‘clock and compass’ in young migrants requires further clarification. It is possible that what is involved is not a detailed map, but a system of beacons based on geomagnetic or other information, which allows first-year migrants to control their position along the migration route, at least to some extent.

### Experiments suggesting the role of magnetic conditions

One of the first dataset that suggested that some first-time migrants need additional non-social information for successful autumn migration and cannot complete it in the basis of innate spatiotemporal program alone came from a study in pied flycatchers *Ficedula hypoleuca* (Beck and Wiltschko, 1988). An earlier study by Gwinner and Wiltschko (1978) demonstrated that garden warblers followed an inborne sequence of directions in captivity without any external stimuli that would suggest that they actually move along their migratory path. However, pied flycatchers studied by Beck and Wiltschko (1988) needed to experience the characteristics of the magnetic field encountered *en route*, in southern Iberian Peninsula and in northern Africa, to display the seasonally appropriate orientation in round arenas. The experiments in garden warblers and pied flycatchers were performed by the members of the same research group, which strengthened the belief that their different outcomes were due to the genuine between-species variation in migratory program, and not to methodological issues.

**FIGURE 9:**
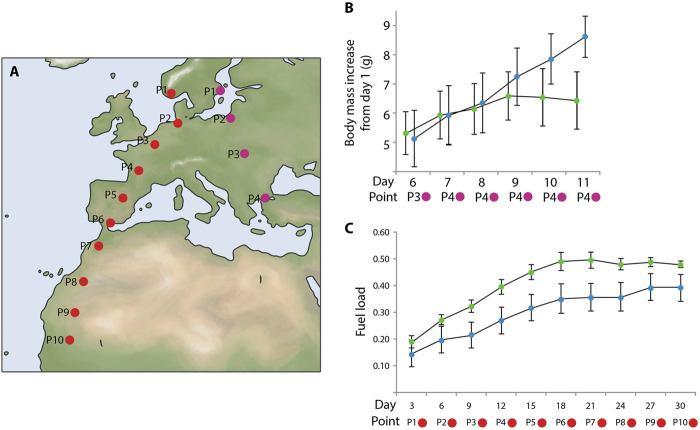
Effect of magnetic-field simulation on migratory refuelling in naïve birds. **(A)** Map showing the geographical locations of the dots used for magnetic simulations for the experimental groups: purple dots–thrush nightingales *Luscinia luscinia*; red dots–northern wheatears *Oenanthe oenanthe*. **(B)** Experimental data from thrush nightingales. Average body mass increase during the experiment: the magnetic field experienced by experimental birds was altered to simulate the conditions of four localities throughout the experiment. Experimental birds were kept in the magnetic field of northern Egypt from day 7 until the end of the experiment on day 11, when all birds were released back into the wild. Green dots: birds consistently exposed to the magnetic field of Sweden. Blue dots: experimental birds exposed to a magnetic field simulating progress along the migratory route. The x-axis represents the days, and the y-axis shows the average body mass increase (g). Error bars represent the standard error. Data for days prior to day 6 are not provided by the authors. **(C)** Experimental data from northern wheatears. Average fuel load, defined as: (body mass - lean body mass)/lean body mass) per 3-day period. Green dots: birds consistently exposed to the magnetic field of northern Germany. Blue dots: experimental birds exposed to a magnetic field simulating progress along the migratory route. The x-axis shows the 3-day periods, and the y-axis shows the average change in fuel load per 3-day period. Error bars represent the standard error. On both graphs, the x-axis shows the days when measurements were taken, as well as the specific points where the experimental birds were located at different times. These points correspond to real geographical areas along the migration route, where the birds might encounter certain magnetic field conditions, such as changes in intensity or direction. For each of these points, similar magnetic field conditions were virtually simulated during the experiment. The points located below the graphs represent these locations on the map, clearly linking the results to actual geographical locations and helping to visualize how the data relates to the birds’ true migration route. This figure is based on the results of Fransson et al., (2001); Bulte et al., (2017).

Another very influential study was published more than a decade later and involved thrush nightingales *Luscinia luscinia* (Fransson et al., 2001; Figures 9A, B). The authors showed that first-time migrants exposed to the magnetic field of northern Egypt, i.e., in front of the major ecological barrier (the Sahara desert), gained significantly more fuel than birds maintained in the magnetic conditions of their capture site (central Sweden). Apparently, thrush nightingales preparing to cross a major barrier need more fuel because refueling opportunities in the desert were extremely limited to non-existent (Biebach, 1990). First-time migrants did not have any prior experience of the magnetic conditions typical of the northern edge of the barrier and could only rely on some innate knowledge when to start serious fueling. Being fat is not without costs for birds, because fatter (i.e., heavier) individuals have less maneuverable flight and are less able to avoid predation (Kullberg et al., 2003; 2007). Even if we keep in mind that the effects of extra fat only start to show when fuel load exceeds ca. 30% of lean mass (Chernetsov, 2012), they are still significant for long-distance flights necessary to cross ecological barriers like the Sahara, the Gulf of Mexico or Central Asian deserts (Bairlein, 1985; Biebach, 1990; Dolnik, 1990; Smolinsky et al., 2013).

Most interestingly, a similar study in northern wheatears *Oenanthe oenanthe* found the opposite effect of simulated magnetic conditions *en route* on fattening (Bulte et al., 2017; Figures 9A, C). Northern wheatears kept in the magnetic conditions that simulated their movement from northern Europe across southwestern Europe to western Africa gained significantly less fuel (not more, as thrush nightingales did) and showed much less nocturnal restlessness than their conspecifics maintained in the magnetic conditions of northwestern Germany. The authors interpreted their results in the sense that wheatears that ‘remained’ in northwestern Germany realized that their perceived position did not fit the position they should occupy in the given season (they were lagging behind their innate temporal schedule) and therefore fueled more and showed more restlessness (were ‘flying’ more) than birds under ‘normal’ conditions (the ones that ‘moved’ as expected).

However, the Fransson et al. (2001) study was replicated. The same research group conducted a similar experiment with the design that included two trials, with 8 birds each (Kullberg et al., 2003). In the early trial (around August 5 ± 2 days) and the late trial (around August 19 ± 2 days) young thrush nightingales captured during the early stage of autumn migration in southeastern Sweden and subjected to magnetic treatment simulating their movement to northern Egypt, accumulated more fuel than birds in the control group. However, in the late trial, regardless of magnetic treatment, the birds had greater fuel reserves, and no difference was observed between the control and experimental groups, unlike in the early trial. The authors suggested that the significance of endogenous and ecological factors for individual birds varies depending on the season and geographical location. As birds approach natural barriers, ecological signals may outweigh the endogenous temporal program and act independently. In the end of the season, being under stricter time constraints, the birds always increase their fuel reserves, which neutralizes the effect of magnetic treatment.

**FIGURE 10:**
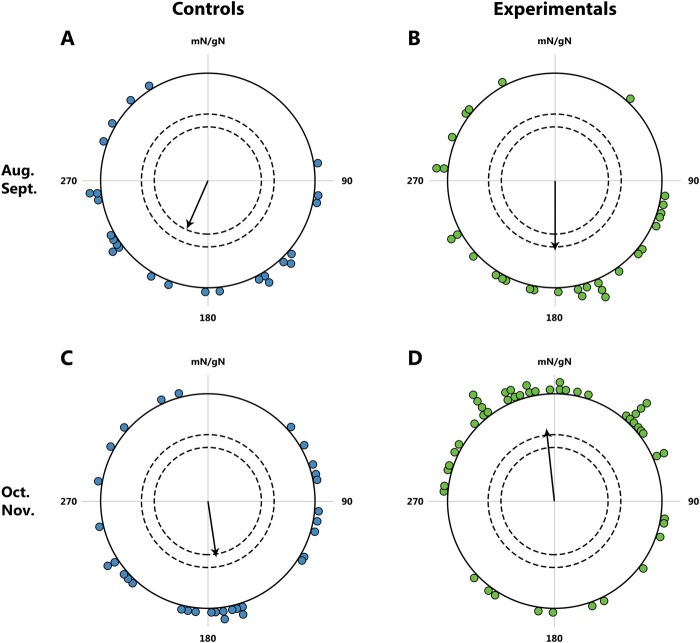
Orientation of garden warblers *Sylvia borin* during autumn migration; tests in the natural geomagnetic field. **(A, B)**: Tests in August and September. Controls (blue dot): N = 25, mean direction 204°, r = 0.43; Experimentals (green dot): N = 30, mean direction 180°, r = 0.44. **(C, D)**: Tests in October and November; beginning on October 1, the experimental birds were exposed to a horizontal magnetic field for 2 days. Controls (blue dot): N = 32, mean direction 169°, r = 0.33; Experimentals (green dot): N = 50, mean direction 353°, r = 0.40. The dots at the periphery of the circle represent the mean heading of a single bird, the arrow represents the group orientation mean vector (circle radius represents a vector length r = 1); the inner and outer dashed circles indicate the required length of r for significance levels of 5% and 1% according to the Rayleigh test, respectively. mN/gN, magnetic North/geographical North. This figure is based on the results of Wiltschko and Wiltschko, (1992).

Another study providing evidence for the existence of innate reference points along birds’ migration routes was conducted by Wiltschko and Wiltschko (1992). The study suggests that birds do not perceive the polarity of the magnetic field (the direction of the field vector), but rather the inclination (the angle between the field lines and the horizontal plane). In this context, particular interest lies in bird species that cross the magnetic equator during migration, where the magnetic field lines run parallel to the Earth’s surface at the magnetic equator, creating an angle of 0° (horizontal magnetic field). In such a situation, distinguishing between the directions ‘toward the equator’ and ‘toward the pole’ becomes impossible (Schwarze et al., 2016). When one such species, the garden warbler, was subjected to laboratory conditions simulating the crossing of the magnetic equator during their autumn migration, 2 days of exposure to a horizontal magnetic field caused a change in their direction from ‘towards the equator’ to ‘towards the pole’, acting as a trigger for significant changes in their migratory behavior (Figure 10). However, a recent experiment with marsh warblers *Acrocephalus palustris* and spotted flycatchers *Muscicapa striata* (Utvenko et al., 2025) did not reveal a shift in orientation direction after exposure to a horizontal magnetic field, suggesting species-specific variation in response to this stimulus.

Robert Beason’s research on bobolinks *Dolichonyx oryzivorus* that breed in central North America and winter in central and western South America, yields valuable data. In his experiments, the birds were tested in a planetarium, where a static star map was shown to them. During the trials, the birds were exposed to a series of alternating artificial magnetic fields that simulated the natural magnetic conditions of the migration route across the equator into the Southern Hemisphere. Despite this, the bobolinks continued to orient southward throughout the experiment. This suggests that the horizontal magnetic field at the equator, possibly in combination with stellar signals, helped them switch from ‘towards the equator’ to ‘towards the pole’ orientation during migration (Beason, 1987; Beason, 1989; Beason, 1992). However, the design of the experiment does not allow for a definitive determination of whether the birds used the magnetic field for orientation. It is possible that they ignored the magnetic field and relied on the star compass they had developed, orienting by the stars displayed in the planetarium.

Despite the significant body of research on the ability of songbirds to perceive the magnetic field (Wynn and Liedvogel, 2023), our understanding of when, where, and how young migrating birds first use magnetic signals to make decisions about migration and orientation in the natural environment remains incomplete. The data obtained from experiments are fragmentary, collected across different bird species, and often not independently verified by research groups other than the authors of the original studies. Independent replication of these experiments in other trans-equatorial migrant species could significantly strengthen the empirical foundation of the research. Furthermore, such studies could shed light on the question of whether there is an innate mechanism that helps young birds in their first year of life to know their position on the migration route.

### The role of photoperiod during the first migration

One of the key factors regulating migration is the photoperiod, which acts as a synchronizer of circannual rhythms and significantly influences the timing of migration (Berthold, 1996; Gwinner, 1996). For example, birds that hatch later in the breeding season and are exposed to shorter day lengths show an accelerated completion of molt and begin migration at an earlier age (Berthold, 1988; Gwinner, 1986; Gwinner, 1989). In contrast, the lengthening days stimulate earlier spring migration and winter molt (Farner and Gwinner, 1980; Gwinner, 1996; Dawson et al., 2001; Rani et al., 2005).

In experiments with long-tailed tits *Aegithalos caudatus*, short daylength stimulated increased locomotor activity, which in the wild would allow the birds to leave northern areas more quickly in the case of delays (Bojarinova and Babushkina, 2015). Studies of closely related chats from three regions, namely, African stonechats *Saxicola axillaris* from East Africa, European stonechats *S. rubicola* from Central Europe, and Siberian stonechats *S. maura* from Siberia raised under naturally varying day lengths, yielded similar results (Helm et al., 2005). Chicks reared under shorter photoperiods, simulating late hatching in late summer, compensated by undergoing accelerated postjuvenile development in ways specific to their populations. Late-hatched individuals from all populations were able largely to catch up with those that hatched earlier, advancing their autumn migratory restlessness (*Zugunruhe*) by 0.9 days for each additional day of later hatching. This genetically programmed compensatory mechanism, supported by field observations of wild individuals, enabled the stonechats to achieve a high degree of synchrony within the population, counteracting the effects of delayed hatching. Similar advancement in migratory restlessness for later-hatched chicks was also observed in birds breeding at low latitudes, such as yellow-green vireos *Vireo flavoviridis* (Styrsky et al., 2004).

Ring recovery analyses in both long- and short-distance migrants have shown that migration speed tends to increase later in the season, with birds migrating later in the season traveling faster than those that depart earlier (Ellegren, 1993; Fransson, 1995; Bensch and Nielsen, 1999; Bojarinova et al., 2008; Bojarinova and Babushkina, 2010; Babushkina and Bojarinova, 2011).

### The role of stars during the first migration

Despite the fact that the star compass is not innate (Emlen, 1967a; Emlen, 1967b), the role of stars in the first migration of birds has been confirmed by a series of experiments conducted under cloudy conditions and with visible starry skies. These experiments indicate the ability of birds to detect and respond to positional changes (compensating for displacement). For instance, in circular arena experiments under the starry sky, birds adjusted for positional shifts, whereas in cloudy weather, their orientation remained unchanged or even reversed (Thorup and Rabøl, 2007). This suggests the possible involvement of stars in detecting positional changes. The role of celestial cues is further emphasized by research conducted under planetarium skies (Beason, 1992).

It is also possible that young birds use stars as beacons during migrations. They could learn and adapt their star compass to specific migration conditions, which could affect their ability to re-route and navigate new geographic environments. Experimental results indicate that the star compass can develop in spring, even after the first migration (Zolotareva et al., 2021). This suggests that the sensitive period for learning it does not end at a specific age. However, the question remains: how flexible is this learning process, and can it continue during migration itself? Can birds re-learn their star compass during migration in response to new conditions?

If so, during migration, birds might observe the starry sky during stopovers for refueling along their route. For example, marsh warblers spend a considerable period in Kenya (Åkesson, 1993b), where they might hypothetically study new for them equatorial star patterns that help them migrate across the magnetic equator. Forming a fully operational star compass may require 2–3 weeks (Michalik et al., 2014; Zolotareva et al., 2021). This highlights the birds’ remarkable ability to quickly learn and adapt to new conditions. The formation and calibration of the star compass are particularly crucial for crossing the magnetic equator, where the inclination magnetic compass malfunctions and becomes ambiguous. The star compass knowledge acquired during a stopover in Kenya enables birds to adapt to changing conditions and confidently continue their migration, guided by celestial cues. This plays a decisive role in the successful completion of their journey.

## Conclusion

The data described above suggest that, in addition to a clock (or calendar) and a compass, inexperienced migrants may possess an innate understanding of the magnetic and/or photoperiodic conditions they are expected to encounter during the typical “on-schedule” passage of their autumn migration route. This innate knowledge may enable them to adjust their migratory behavior in the event of deviations from this schedule. Furthermore, birds may recognize changing conditions during migration, learn from them, and use additional information to adjust their migration routes.

We suggest that the data currently available warrant a revision of the clock and compass concept. It seems that young individuals of many migratory species are aware of their position along the migration route with varying degrees of accuracy. Below, we present our speculations on how young birds undertaking their first autumn migration from the Northern Hemisphere to the Southern Hemisphere might employ a variety of navigation mechanisms, which change depending on the stage of their journey.

At the initial stage, when leaving their birthplace, birds rely on cues learned during natal dispersal. These cues may include odors remembered from their native area (Gagliardo, 2013) and visual features of the landscape, such as characteristic contours of the terrain or water bodies.

The innate magnetic compass and the star compass, if learned and established, allow birds to choose the general direction of migration. As birds move further away from their native territory, they begin to use additional mechanisms. Among these, genetically encoded navigation beacons related to Earth’s magnetic field parameters may play a significant role. Changes in magnetic inclination or intensity before major ecological barriers can serve as landmarks, helping birds refine their path (Fransson et al., 2001; Bulte et al., 2017). Additionally, some species may have an innate ability to reorient upon encountering the horizontal magnetic field of the equator, where the magnetic field lines become nearly parallel to Earth’s surface (Wiltschko and Wiltschko, 1992).

It is interesting to hypothesize that birds engaged in trans-equatorial migration, upon stopping near the equator, are able to retrain their star compass, adapting it to the stellar patterns of the Southern Hemisphere. This could allow them to continue their journey through the magnetic equator where the magnetic compass becomes ineffective (Schwarze et al., 2016). However, this hypothesis requires further research, as there is currently insufficient evidence to confirm that birds can adapt their star compass in response to hemisphere shifts.

In the final phase of migration, as birds approach their wintering grounds, they begin to rely again on innate landmarks. Genetic patterns may guide them to specific habitats suitable for wintering. Throughout migration, birds can track the number of days spent in particular areas, enabling them to align their movement with their internal biological clocks and adjust their speed based on their current location. This ability, known as the “clock and compass” concept, complements their suite of navigation strategies, ensuring a successful completion of their first journey. Such a complex set of mechanisms allows young birds to cover vast distances and reach the Southern Hemisphere despite their lack of experience.

Further research should focus on the sources of information they rely on and the precision of this control.
